# A Zero-Shot Low Light Image Enhancement Method Integrating Gating Mechanism

**DOI:** 10.3390/s23167306

**Published:** 2023-08-21

**Authors:** Junhao Tian, Jianwei Zhang

**Affiliations:** School of Computer Science, Sichuan University, Chengdu 610065, China; swewjk@gmail.com

**Keywords:** low light enhancement, zero-shot, transformer, lightweight

## Abstract

Photographs taken under harsh ambient lighting can suffer from a number of image quality degradation phenomena due to insufficient exposure. These include reduced brightness, loss of transfer information, noise, and color distortion. In order to solve the above problems, researchers have proposed many deep learning-based methods to improve the illumination of images. However, most existing methods face the problem of difficulty in obtaining paired training data. In this context, a zero-reference image enhancement network for low light conditions is proposed in this paper. First, the improved Encoder-Decoder structure is used to extract image features to generate feature maps and generate the parameter matrix of the enhancement factor from the feature maps. Then, the enhancement curve is constructed using the parameter matrix. The image is iteratively enhanced using the enhancement curve and the enhancement parameters. Second, the unsupervised algorithm needs to design an image non-reference loss function in training. Four non-reference loss functions are introduced to train the parameter estimation network. Experiments on several datasets with only low-light images show that the proposed network has improved performance compared with other methods in NIQE, PIQE, and BRISQUE non-reference evaluation index, and ablation experiments are carried out for key parts, which proves the effectiveness of this method. At the same time, the performance data of the method on PC devices and mobile devices are investigated, and the experimental analysis is given. This proves the feasibility of the method in this paper in practical application.

## 1. Introduction

Due to the limitations of the technical environment and technology, ambient light affects the quality of the photos taken by the camera. This leads to the problems of reduced image quality, reduced viewing distance, and reduced effective information contained in the images. Reduced illumination in the image leads to reduced model accuracy in target tracking and detection. Low-light image enhancement plays a very important role in image processing and machine vision. However, at present, most AL algorithms rely on pairs of training data to train the image-to-image mapping relationship. These data sets are difficult to obtain in real scenes. Therefore, the non-reference low light image enhancement algorithm has a high research value and is challenging.

Many deep learning methods have been proposed to deal with low light enhancement problems. LPNet [1] introduced the Gaussian-Laplacian image pyramid decomposition technique, which enhanced the effectiveness of low-light image enhancement methods. In Retinex-Net [2], the Retinex theory was used to decompose the image, which reduced the noise while enhancing the brightness of the image. CycleGAN [3] utilizes the GAN (Generative Adversarial Network) approach for cycle-consistent supervision, which enhances the effectiveness of image mapping learning. However, these methods have a major drawback, which is the need for a significant amount of effort to collect or create a sufficient number of paired training data. Additionally, the quality of these training data greatly affects the performance of the algorithm. Moreover, the existing methods mostly rely on learning the mapping relationship between images, but this approach heavily depends on the training images and may not achieve satisfactory restoration results when there is a significant disparity between the image scene and the training images. To address this issue, the main focus of this research is unsupervised low-light image enhancement methods (also known as zero-shot low-light image enhancement methods), which aim to enhance the illumination of images without paired low-light image data.

In this paper, we propose a method based on deep learning, which can cope with various conditions of images, such as illumination conditions, including uneven illumination and poor brightness. Different from the method of learning image-to-image mapping, in order to avoid the model relying too much on paired images, we use the method of estimating the parameters of the enhancement curve to enhance it. We design a differentiable curve and use deep learning technology to learn the adjustable parameters of the curve.

**Figure 1:**
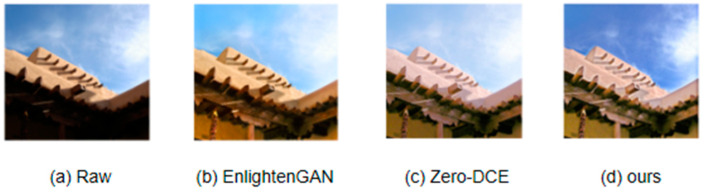
As an example, it can be seen from the image that the enhancement effect of the method in this article is compared with the other two existing methods on the same image. It can be seen that the method in this article has a better effect on enhancing the overall brightness of the image.

Figure 1 is a small example of low-light enhancement. We have selected one of the current two mainstream methods and compared its results with the methods in this article. EnlightenGAN [4] uses GAN (Generative Adversarial Networks)’s [5] method for low-light image enhancement, which has more obvious advantages compared to end-to-end methods. At the same time, the supervised method has a larger model volume and stronger image supervision information compared to this method. From the above figure, it can be seen that the rendering of this method has better restoration and visual effects compared to the other two methods in terms of image quality. Due to the end-to-end training approach adopted in this method, compared to the other two methods, this method may have shortcomings in restoring some details of the image. This is because other methods have improved the restoration effect of local areas after adding corresponding modules. However, from the graph, it can be seen that the method proposed in this article can still produce good image restoration results. The reason for the brighter image effect of this method is that due to the lack of contrasting ground truth images, the overall brightness of the image will become brighter.

The contributions of this article are as follows:

1. For unsupervised low-light image enhancement tasks, this article proposes a solution that incorporates intermediate images to enhance the illumination of the image through a two-stage method.
2. We design a set of Loss functions for zero sample low light image enhancement and prove their effectiveness.
3. We design a parameter grouping enhancement method to enhance the robustness of the network model in low-light image enhancement tasks.

## 2. Related Work

### 2.1. Low Light Enhancement

In the past decades, scholars have proposed many low light image enhancement algorithms, which mainly go through three stages: histogram equalization, Retinex theory, and convolutional neural network.

Histogram equalization is the most classic image enhancement algorithm in the early days. This algorithm calculates the frequency of input image pixels so that the histogram of input image pixels conforms to the distribution, thus improving the image contrast. This type of method is easy to implement, but it ignores the excessive enhancement caused by local brightness differences in the image and generates strong noise.

The second stage is an algorithm based on Retinex theory, which believes that the color of an object is not affected by light and has color constancy. Therefore, the image is decomposed into two parts: the illumination component and the reflection component. Among them, the reflection component is the intrinsic property of an object that is independent of lighting conditions, and the lighting component can reflect the difference between low light and normal light. The algorithm based on this theory first estimates the illumination component of low-light images and then enhances them to obtain corresponding normal-light images. The single-scale Retinex algorithm [6] is the first low-light image enhancement algorithm based on Retinex theory, which ensures the smoothness of the illumination image through a Gaussian filter. The next multi-scale Retinex algorithm with color restoration [7] is improved on the basis of single-scale SSR, which uses a Gaussian filter of different scales and performs color restoration. In recent years, many low-light image enhancement algorithms combined with Retinex theory have been proposed. NPE [8] uses logarithmic transformation and Retinex theory to jointly enhance image contrast and maintain the naturalness of image illumination; SRIE [9] proposed a weighted variational model to estimate both reflection and illumination components simultaneously; LIME [10], on the other hand, only estimates the illumination component and outputs the reflection component as the enhancement result; MF estimates illumination components based on closed operations in morphology, and fuses multiple images for low light image enhancement. Although the above algorithms have achieved good results in certain scenarios, they cannot overcome the natural drawbacks of the Retinex theory. Their performance is limited by the model’s ability to decompose reflection and illumination components, and sometimes manual parameter adjustments are required, resulting in weak pan-Chinese ability. Moreover, most of these methods overlook noise and color distortion.

The emergence of deep learning and convolutional neural networks has greatly promoted the development of image restoration technology. Retinex Net [2] combines Retinex theory with deep learning. MBLLEN [11] achieves low illumination image enhancement and maintains image quality through feature fusion. DeepUPE [12] first estimates the brightness map of the image and designs a special brightness loss function to adjust brightness. EnlightenGAN [4] and Zero DCE [13] take a different approach in the case of not using paired data for training.

### 2.2. Vision Transformers

The application of Transformers [14] in computer vision and low-level image processing has grown. Many Researchers’ innate attention mechanism, which gives them the ability to recognize long-term dependencies in the data, is the key to their success. Transformers have recently shown amazing performance in computer vision tasks, particularly in picture classification [15,16,17], segmentation [17,18], and object detection [19,20]. Many researchers have begun employing transformers for these tasks.

To allow transformers to handle 2D images, an input image $I\in R^{H\times W\times C}$ is divided into non-overlapping patches of size (P, P). Each patch is flattened and projected to a d-dimensional vector via a trainable linear projection, forming the patch embeddings $X\in R^{N\times d}$ where H, W are the height and width of the image, respectively, C is the number of channels, and $N=H\times W/P^{2}$ is the total number of patches. Finally, N is the effective input sequence length for the transformer encoder. Patch embeddings are enhanced with position embeddings to retain 2D image positional information.

In [14], the authors design a new vision transformer encoder by stacking blocks of multi-head self-attention (MSA) and MLP layers. To solve the problem of gradient explosion or gradient dispersion, a residual mechanism is applied after every block, $X\in R^{N\times d}$ with dimension d as input; an MSA block produces an output sequence $\tilde{X}=R^{N\times d}$ via

(1) $$Q=XW_{Q},K=XW_{K},V=XW_{V}\;A=Softmax(QK^{⊺}/\sqrt{d})\;\bar{X}=AV$$

where $W_{Q}$, $W_{K}$ and $W_{V}$ are d×d learnable matrices. Then it transforms the sequence X to keys, queries, and values, respectively.$\tilde{X}$ is a linear combination of all the values in V weighted by the attention matrix A. In turn, A is calculated from similarities between the keys and query vectors.

Transformers compute self-attention A and use that information $\bar{X}$ to build models. Self-attention cannot be applied directly to images as N since it quickly becomes uncontrollable due to its quadratic cost in time and space. Due to this intrinsic limitation, modality-aware sequence length constraints have been used to limit sequence length while preserving model performance. A transformer design can be applied directly to medium-sized image patches for various vision applications, as demonstrated in reference [15]. This local self-attention reduces the aforementioned memory constraints.

In low-level vision areas, transformer-based models have also made much progress on several sub-directions, such as image super-resolution [21], image restoration [22,23], image colorization [24], and bad weather restoration [25]. Very recently, MAXIM [26] used an MLPbased model in low-level vision areas which also shows MLP’s potential on low-level vision tasks. However, existing transformer & MLP models require much computational cost (e.g., 115.63M for IPT [27], 14.14M for MAXIM [26]), making it hard to implement on mobile and edge devices. Similarly, its application in the field of low-light image enhancement often incurs significant model overhead, and the method model proposed in this article can effectively solve this problem.

## 3. Method

### 3.1. Motivation

In traditional image processing, it is known that the general process of image restoration is the mapping between the pixels of the input image $I_{i}$ and the restored image $I_{R}$, which can be represented by the following formula:

(2) $$I_{R}=N(I_{i}),$$

Generally speaking, the features of an image are extracted first through a shallow feature extractor to initially extract the detailed information in the image, which is usually implemented by a single convolution layer with a convolution kernel size of 3. After extracting the preliminary shallow information of the image, the image detail information is extracted from the original image by stacking convolutional layers. In the method proposed in this paper, we introduce the Transformer structure to enhance the performance of feature extraction. It uses a finer-grained feature extraction process to better extract the features of the image, and at the same time, it can also perform better for many details of the image it deals with.

In previous experiments, we found that simply using stacked convolutional layers or Transformer layers can lead to the recovery of some useless image details in the image feature map during the recovery process. In this article, we used a gating mechanism to filter out useless information, which is different from other methods where we use the hardware function for processing, it can filter out some useless details in the extracted image features. In addition, to address the issue of convolutional layers not being able to effectively process images, we have also adopted a Transformer structure to enhance the feature extraction ability of the image. At the same time, we have also reduced the size of the multi-head attention module in the network module, reducing the size of the model and improving processing speed.

### 3.2. Model Structure

**Figure 2:**
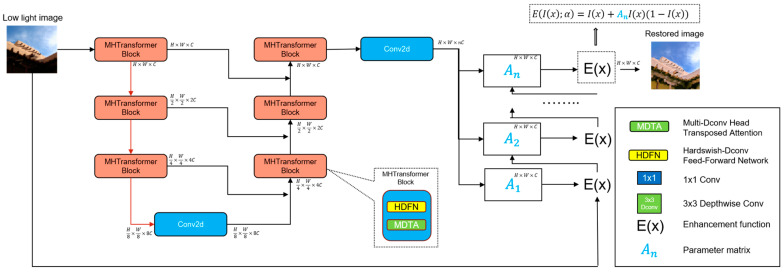
The structure of our proposed network. The network includes an image feature extraction net and an iterative enhancement net.

Figure 2 shows the pipeline of our architecture proposed in this article, which consists of two parts: (i) the image feature extraction stage and (ii) the iterative enhancement stage. This method first takes low-light images as input and uses the network output as parameters for each iteration of enhancement. By training the image feature extraction network, it can output the best results. At the same time, in order to improve the ability of image feature extraction, we introduced the Transformer structure and introduced a new loss function to ensure that the image recovered from the network will not have sudden changes in pixel brightness. We also introduced new HDFN modules and modules to improve the network performance.

### 3.3. Image Feature Extraction Net

The function of an image feature extraction network is to extract the detailed features of the image from the original image features through a neural network and convert them into enhancement parameters. The overall network adopts an encoder-decoder structure, in which the convolutional layers in the original architecture are replaced with the proposed MHTransformer module introduced in this paper. This replacement aims to enhance the network’s feature extraction capability for the original low-light images, ultimately generating higher-quality parameter matrices.

*MHTransformer* This article introduces the Transformer module to enhance the detailed feature extraction function of the model. As usual, the structure in this article also includes two parts: a multi-head attention stage and a forward propagation stage. In order to better preserve the detailed features of the image while removing useless information from the detailed features, this article designs an MDTA module and an HDFN module.

*MDTA* In the Transformer structure, the main computational burden comes from the self-attention mechanism layer. Due to this process, there are usually three steps required for processing.

Given a Query and a series of Key-Value pairs, map an output together. This includes three steps:

- Measure the similarity between Query and Key
- Scaling and standardizing the obtained similarity measure
- Weighting weights with Value

**Figure 3:**
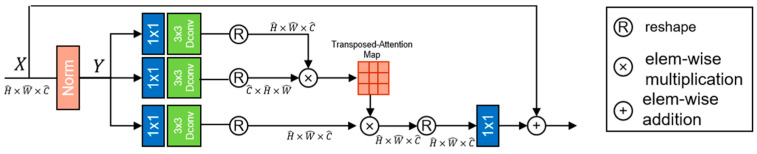
The structure of MDTA.

In this process, it will bring huge computational overhead to the entire network, and when the self-attention mechanism is applied to high-resolution images, this problem becomes increasingly serious, seriously affecting its application in the field of image restoration. To address this issue, we introduced MDTA, which has linear complexity. The key to this structure’s linear complexity is that we applied the self-attention mechanism (SA) to the channel dimension rather than the spatial dimension. Therefore, we introduced MDTA with linear complexity here, and also introduced deep separable convolutions into the structure. After obtaining a layer normalization tensor, the module first propagates forward to generate query (Q), key (K), and value (V). By applying 1 × 1 convolution to aggregate pixel and cross channel contexts, and applying 3 × 3 deep convolution to encode channel level spatial contexts, the module generates $Q=W_{p}^{Q}W_{d}^{Q}Y$, $K=W_{p}^{K}W_{d}^{K}Y$, $V=W_{p}^{V}W_{d}^{V}Y$. Among $W_{p}^{\left(\cdot \right)}$ is 1×1 point by point convolution and $W_{d}^{\left(\cdot \right)}$ is 3×3 deep convolution. Overall, the module process is defined as

(3) $$\overset{\wedge }{X}=W_{p}Attention\left(\overset{\wedge }{Q},\overset{\wedge }{K},\overset{\wedge }{V}\right)+X,\;$$

(4) $$Attention(\overset{\wedge }{Q},\overset{\wedge }{K},\overset{\wedge }{V})=\overset{\wedge }{V}\cdot Soft\max(\overset{\wedge }{K}\cdot \overset{\wedge }{Q}/\alpha ),\;$$

where X and $\hat{X}$ are input and output feature maps; A matrix $\overset{\wedge }{Q}\in R^{\overset{\wedge }{H}\overset{\wedge }{W}\times \overset{\wedge }{C}}$, $\overset{\wedge }{K}\in R^{\overset{\wedge }{C}\times \overset{\wedge }{H}\overset{\wedge }{W}}$, $\overset{\wedge }{V}\in R^{\overset{\wedge }{H}\overset{\wedge }{W}\times \overset{\wedge }{C}}$ is obtained by modifying a tensor from its original size.The MDTA structure is shown in Figure 3.

*HDFN* The ordinary forward propagation module operates on the values at each pixel position. It uses two convolutions, one to extend the feature channel and the second to reduce the channel back to the original input dimension. The HDFN structure is shown in the figure above. We hope to filter out the required information through a gating mechanism. In this architecture, the gating mechanism is formalized as the product of elements of two parallel paths in the linear transformation layer, and one of them is activated using Hardswish [28]. At the same time, we also use deep separable convolution in the network to encode information from adjacent pixel positions in space, which is very useful in many image restoration tasks.

**Figure 4:**
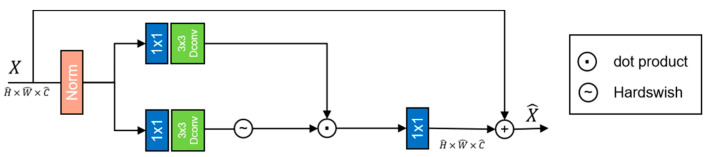
The structure of HDFN.

Given an input tensor $X\in R^{\overset{\wedge }{H}\times \overset{\wedge }{W}\times \overset{\wedge }{C}}$, HDFN is formalized as follows:

(5) $$\overset{\wedge }{X}=W_{p}^{0}Gating\left(X\right)+X,$$

(6) $$Gating\left(X\right)=\phi \left(W_{d}^{1}W_{p}^{1}\left(LN\left(X\right)\right)\right)⊙W_{d}^{2}W_{p}^{2}\left(LN\left(X\right)\right),\;\;$$

wherein, ⊙ represents the element by element multiplication, ϕ represents the Hardswish activation function, and LN is the layer normalization. The HDFN structure is shown in Figure 4.

### 3.4. Iterative Enhancement Net

After extracting image detail features from the original low-light image and converting them into an enhancement parameter matrix, it is necessary to use the parameter matrix to enhance the image pixel by pixel. In order to make the enhancement network in this paper better adapt to different image environments, a parameter matrix block enhancement method is designed in this paper, which can better cope with various lighting conditions in the image.

*Enhancement function* Inspired by traditional image processing methods that focus on pixel-to-pixel enhancement, this paper references their approach and designs an enhancement function for mapping pixels from the original low-light image to the pixels in the enhanced image with improved illumination. In order to make the image enhancement function effective, the function should have the following two characteristics: (1) The image is a high-order curve that can better simulate the mapping relationship between image pixels. (2) The image should be smooth and differentiable, which can ensure the quality and smoothness of lighting after image restoration. The function designed in this article is as follows:

(7) $$E(I(x);A_{n})=I(x)+A_{n}I(x)(1-I(x)),$$

In the function, $A_{n}$ represents the parameters estimated by the network and n indicates multiple enhancement stages. In this study, the same enhancement curve is used in multiple stages but with different parameters. This is done to better adapt to various low-light scenes in the image during the network enhancement process in order to achieve the best enhancement effects.

**Figure 5:**
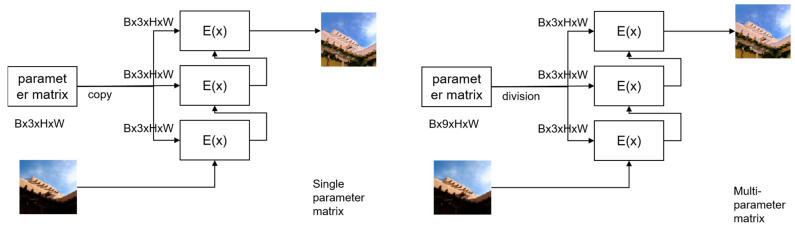
The comparative graph of the two enhancement methods is shown below. One method uses a single parameter matrix, while the other method divides the parameter matrix into blocks for enhancing different image regions in multiple stages.

*Parameter matrix block enhancement method* The existing multi-stage enhancement methods use the same module and parameters for enhancement. The disadvantage of this enhancement method is that for some images with uneven lighting, the same model and parameters can cause excessive enhancement of the image. To solve this problem, this article proposes a parameter matrix block enhancement method. The advantage of dividing the estimated parameter matrix into blocks and applying it to each enhancement is that during the training process of the neural network, the network can adapt to different circumstances. The comparison between the two methods is illustrated in Figure 5:

As shown in Figure 5, the parameter matrix is incorporated into the enhancement curve on a pixel-to-pixel basis, and the calculations are performed using the curve. In the figure, E(x) represents the $E(I(x);A_{n})$ mentioned earlier in the text.

### 3.5. Loss Function

As an unsupervised method, this method needs to introduce an unsupervised loss function for network training due to the lack of supervision of paired images during training. Regarding the images of the restored illumination obtained in the two stages mentioned earlier, this paper calculates their respective loss function values. Subsequently, these values are employed to train and optimize the entire network model, resulting in the attainment of a well-optimized network model. The five loss function introduced in this method will be introduced below.

*Spatial loss* In the process of enhancing lighting, the pre-enhanced and post-enhanced images should have spatial consistency, and the loss of spatial consistency is mainly used to ensure the spatial consistency of the enhanced image, mainly by ensuring the difference in adjacent areas between the original image and the enhanced image.

(8) $$L_{spa}=\frac{1}{K}\sum_{i=1}^{K}\sum_{j\in \Omega (i)}(\left|Y_{i}-Y_{j}\right|-\left|I_{i}-I_{j}\right|{)}^{2},\;\;\;\;\;$$

where K is the number of local regions and is the four adjacent regions of region i (top, bottom, left, and right). We define Y and use it as the average intensity value of the local area of the enhanced image. For the size of the local area, first set it to 4×4.

*Exposure controller loss function* In order to solve this problem, the exposure control loss function is introduced to suppress these areas. Specifically, the average intensity value under good exposure is first defined. Then for each local area, the difference between its average intensity value and the average intensity value under good exposure is calculated. For the average intensity value of good lighting, set it to 0.6. The exposure control loss can be expressed using the following formula.

(9) $$L_{exp}=\frac{1}{M}\sum_{k=1}^{M}\left|Y_{k}-E\right|,$$

In the formula, M represents the number of non-overlapping regions in the image (using small regions for non-overlapping segmentation), and Y is the average intensity value of local regions in the enhanced image.

*Color consistency loss* In order to make the color of the enhanced image visually acceptable, a color consistency loss function is designed to control the overall color of the image. From the formula, we can see that the pixel contrast formula is as follows:

(10) $$\;\;\;\;\;\;L_{col}=\sum_{\forall (p,q)\in e}(J^{p}-J^{q}),\varepsilon =\{(R,G),(R,B),(G,B)\},$$

The formula $J^{p}$ represents the average intensity value of the p channels in the enhanced image, and $J^{q}$ represents the average intensity value of the q channels.

*Light smoothing loss* In order to maintain the monotonic relationship between adjacent pixels, lighting smoothing loss is introduced, and the formula is as follows:

(11) $$L_{ls}=\frac{1}{N}\sum_{n=1}^{N}\sum_{c\in \xi }(|\nabla_{x}A_{n}^{c}|+|\nabla_{y}A_{n}^{c}|{)}^{2},$$

*TV loss* Generally speaking, there are many image quality issues in dark images, with the most significant being the impact of image noise. Therefore, in this chapter, total variational loss is introduced to suppress noise in the image.

(12) $$L_{tv}=\frac{1}{CHW}\sum_{c=1}^{C}\sum_{h=1}^{H}\sum_{w=1}^{W}[(\nabla_{x}Y_{c,h,w}{)}^{2}+(\nabla_{y}Y_{c,h,w}{)}^{2}],$$

*Total loss* The formula of the total Loss function is as follows.

(13) $$L_{total}=\lambda_{spa}\cdot L_{spa}+\lambda_{exp}\cdot L_{exp}+\lambda_{col}\cdot L_{col}+\lambda_{ls}\cdot L_{ls}+\lambda_{tv}\cdot L_{tv,}$$

wherein $\lambda_{spa}$, $\lambda_{exp}$, $\;\lambda_{col}$, $\lambda_{ls}$ and $\lambda_{tv}$ is the weight parameter.

## 4. Experiments

In order to demonstrate the superiority of this method in unsupervised low-light image enhancement tasks, experiments were conducted, and the results were compared with current advanced low-light image enhancement algorithms. During the experiment, the algorithm was implemented using the Python framework, with a single RTX3090 graphics card as the GPU.

### 4.1. Experimental Setting

*Datasets* Typically, CNN-based methods require paired datasets for operation, while GAN-based methods require carefully selected non-paired training data. In this task, it is necessary to estimate the curve parameters from the input images. Therefore, when selecting training data, images with different exposure levels were selected to enhance the robustness of this method. This also proves that our proposed method has good adaptability to low-light images. So we chose to select images with different exposure levels from the first part of the SICE dataset [29] and segment them as training and validation sets to train our proposed network. To validate the effectiveness of the proposed method in this paper, a series of experiments were conducted using five publicly available benchmark datasets DICM [30], LIME [10], VV [31], MEF [32], and Fusion [15] specifically designed for low-light image enhancement. These datasets exclusively consist of low-light images without their corresponding normal-light counterparts. The reason behind selecting these datasets is that comparing only the restoration quality metrics would be insufficient for evaluating the performance of low-light image enhancement. Therefore, in this study, a combination of metric comparisons and visual effect demonstrations was employed to comprehensively evaluate the experimental results and demonstrate the efficacy of the proposed method. 

*Hyperparameters* The training batch size used in the experiment is 2, the validation batch size is 4, the initial learning rate is 0.0001, and the weight attenuation is 0.0001.

*Training and inference strategies* In this experiment, the non-reference loss function is directly used to train the network. In order to compare the effect of this method and other comparison methods, reasoning is carried out at the same resolution.

### 4.2. Experimental Evaluation Indicators

Due to the lack of corresponding images of normal lighting in the test set used in the experiment, the NIQE [33], PIQE [34], and BRISQUE [35] evaluation index is used for comparison of experimental results.

In addition, in low-light image enhancement tasks, the superiority of visual effects is more important than metric performance. The reason this paper uses these three metrics instead of PSNR [36] or SSIM [37] is that these three metrics can better evaluate the visual effects of images, avoiding the situation where the low-light enhancement results are affected by the poor quality of the reference image.

### 4.3. Comparison with Other Methods

**Figure 6:**
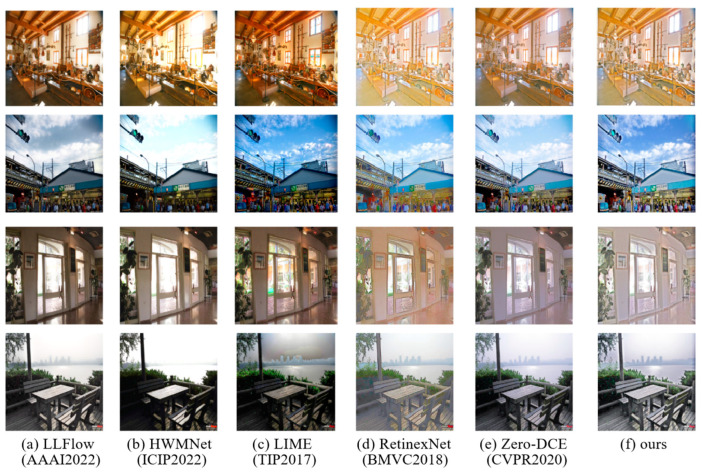
Compared with other existing methods in terms of visual effects, it can be seen that this method has the best effect in enhancing the overall brightness of the image compared to other methods.

**Figure 7:**
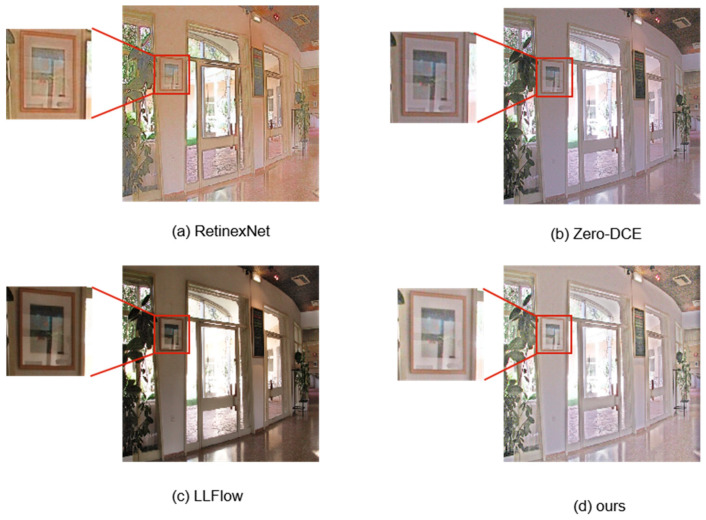
In terms of the comparison of the method in this paper with other methods on the details, it can be seen that the proposed method in this paper performs well in terms of brightness and detail preservation compared to other methods.

In order to demonstrate the effectiveness of this method compared to other methods, this article compared it with other methods on several datasets. Due to the lack of corresponding normal lighting data in the selected test dataset, all tests in this article used single image evaluation indicators for performance comparison. For the selection of other methods for comparison, as the research object in this article is unsupervised methods, which have disadvantages compared to supervised methods, several representative supervised/unsupervised methods have been selected here. The comparison of visualization results and indicator results is shown in Figure 6 and Table 1. In addition, a comparative analysis of the method’s performance in terms of details was also conducted, as shown in Figure 7.

### 4.4. Speed Test

As mentioned earlier, one advantage of the proposed method in this article is the small size of the model and fast inference speed. Many personal applications have migrated from traditional desktop platforms to mobile platforms. Therefore, it is necessary to explore the application of deep learning on mobile devices and embedded platforms. In order to verify the advantages of this article, this article used the method proposed in this article to test on the same test set on the desktop; the desktop device is equipped with an AMD Ryzen 5600X CPU, 24GB of RAM, and an NVIDIA RTX 3060 GPU, then we calculated the inference speed. The image resolution used for inference is 512 × 512 pixels in all cases. The test results are given in Table 2.

From the above table, it can be seen that this experiment can still approach real-time (24FPS) after introducing a Transformer based structure with good inference performance and application prospects.

In addition to conducting inference speed testing on the PC end, speed testing was also conducted on the mobile end. Unlike the PC end, due to the limitations of the mobile code library, images cannot be uniformly sized and cropped. Therefore, experiments were only conducted on the original resolution of each dataset, but this did not affect the analysis of the experimental results. The mobile platform used for inference is Honor 30S; it is equipped with a Kirin 820 (up to 2.36 GHz) processor and 8 GB of RAM. The image resolution used for inference is 640 × 640 pixels in all cases. The test results are given in Table 3.

From the experimental results, it can be observed that the proposed method in this paper achieves an inference time of under 1 s on mobile devices, making it highly promising for applications on devices with limited memory and computational resources.

In addition, it is worth mentioning that the model proposed in this paper has a size of only 99 M, even after incorporating Transformer structures. This presents an unparalleled advantage compared to other methods, such as HWMNet, which has a size close to 700 M. Given the limited storage capacity of mobile devices, this advantage is remarkable. Future research will also consider further exploring the applicability of the proposed model.

### 4.5. Ablation Experiment

In this section, regarding the key component designed in this paper as mentioned, in order to demonstrate the improvement in low-light image illumination restoration, an ablation experiment is conducted specifically on the proposed improvement part in this paper, affirming its effectiveness.

**Figure 8:**
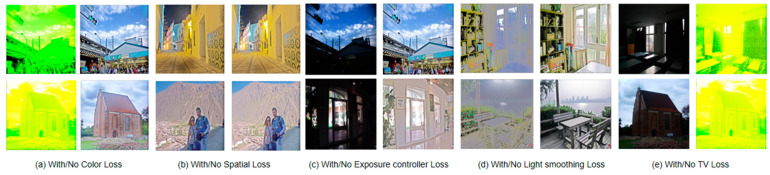
The Influence of Loss Functions on Experimental Results in Visual Effects. It can be seen that the Loss function designed in this paper has played a role in improving image quality.

*Influence of Loss function on indicators and visual effects* In order to verify the effect of several loss functions introduced by the method in this paper, the following are the visual effects and data indicators of the effect of the loss function. The comparison results are shown in Figure 8 and Table 4.

*Ablation result of enhancement method* As mentioned earlier, a new parameter enhancement method was proposed in this article. In order to further validate the effectiveness of the method, ablation experiments were conducted under the same experimental conditions, and the experimental indicators (NIQE) results are shown in Table 5.

From the above table, it can be seen that the parameter-blocking enhancement method proposed in this article has significantly improved the lighting restoration effect of images.

*The Impact of Gating Mechanism Selection* In the past deep learning practice, there were generally three choices of Activation function: Relu, Gelu, and Hardswish, which can be seen from the image is the selection of the hardswish Activation function is more effective for filtering useless information. In order to show the effect of the selected Activation function more clearly, ablation experiments on recovery indicators were also conducted for the selection of the Activation function. The experimental indicators (NIQE) results are shown in Table 6.

From the above table, we can see that the gating mechanism of the hardswish Activation function used in this paper can effectively filter out the useless information in the image features and improve the image illumination enhancement effect.

### 4.6. Superiority and Limitation

In the previous discussion, this paper analyzed the key structures in the method and presented the experimental results. It can be seen that the proposed method in this paper has several advantages compared to previous methods:

- Compared to previous methods, this paper has a significant advantage in terms of model size. It can be effectively deployed on edge devices
- An effective zero-shot low-light enhancement approach is proposed in this paper, which can effectively enhance low-light images without the need for paired training datasets

However, the method proposed in this paper still has the following two limitations:

- Due to the limitations of the zero reference method, this paper is still not very effective in preserving some of the detailed features of the image.
- As this paper uses estimated enhancement parameters for light enhancement of the image, the overall brightness of the image will be on the light side.

## 5. Conclusions

To address the problems of excessive model inference speed in low-light image enhancement tasks and the inability to obtain paired training datasets in real-world scenarios, an unsupervised low-light image enhancement method is proposed in this paper. A two-stage low light image enhancement network is also proposed to address the shortcomings of previous single-stage networks by proposing the concept of intermediate graphs to extract detailed features from input images. The test results on the test set indicate that the proposed method has better recovery performance compared to other existing unsupervised methods. The good applicability of the proposed method has also been verified by experiments in this paper.

## 6. Future Works

For the zero-shot low-light image enhancement method studied in this paper, the core issue is how to evaluate image quality and calculate the loss function to optimize the network in the absence of reference images. We have noticed that besides the intrinsic features of the image itself, there is other useful information, such as semantic information and classification information, that can be utilized. In future research, these pieces of information can be employed to supervise the network and further improve the authenticity of image restoration.

## Figures and Tables

**Table 1:** Comparison of indicators between this method and other existing methods.

Dataset	Method	DICM	Fusion	VV	LIME	MEF
BRISQUE	RetinexNet [2]	60.8657	46.5634	47.2645	57.6542	64.6348
	LIME [10]	18.5645	25.6459	16.9940	23.6523	36.5751
	HWMNet [38]	45.1278	40.3643	43.8894	50.7362	59.7914
	LLFlow [39]	47.1412	44.7718	36.5643	47.5768	57.7648
	Zero-DCE [13]	38.3423	38.2709	23.3306	38.9009	45.6532
	ours	**16.4563**	**24.6827**	**13.6759**	**18.3549**	**34.9531**
PIQE	RetinexNet	14.2645	13.4467	13.4863	23.7415	18.6634
	LIME	9.0064	8.1246	8.6423	13.7756	11.6124
	HWMNet	11.5547	10.5478	9.8874	16.3248	14.2364
	LLFlow	13.5468	11.2468	10.6547	18.6654	15.6478
	Zero-DCE	9.1191	8.4074	7.5945	14.0651	12.3337
	ours	**8.5136**	**7.6112**	**7.2354**	**13.2569**	**11.0056**
NIQE	RetinexNet	7.4727	6.5544	7.2204	7.2982	7.9368
	LIME	5.2545	4.5939	6.8150	6.5213	6.7321
	HWMNet	5.9278	4.3693	5.7690	6.7902	6.7899
	LLFlow	7.0412	4.2728	6.5043	7.5338	7.6948
	Zero-DCE	5.7646	4.1892	5.9308	6.5220	6.1791
	ours	**5.1007**	**4.1534**	**5.4694**	**6.0549**	**5.5248**

**Table 2:** Average inference time for each dataset on PC station.

Datasets	DICM	VV	Fusion	MEF	LIME
time	43.2 ms	43.7 ms	44.1 ms	42.2 ms	48.1 ms

**Table 3:** Average inference time for each dataset on mobile station.

Datasets	DICM	VV	Fusion	MEF	LIME
time	609 ms	578 ms	749 ms	842 ms	715 ms

**Table 4:** Experimental indicator results of ablation of Loss functions.

Loss	NIQE	Fusion	Fusion
Color consistency loss	No color Loss	5.4869	6.0771
	With color Loss	**5.4694**	**6.0549**
Light smoothing loss	No TVA Loss	48.4421	50.9792
	With TVA Loss	**5.4694**	**6.0549**
Exposure controller loss	No exp Loss	8.3966	15.5958
	With exp Loss	**5.4694**	**6.0549**
Spatial loss	No spa Loss	5.6511	6.8697
	With spa Loss	**5.4694**	**6.0549**
TV loss	No TV Loss	6.1443	6.8183
	With TV Loss	**5.4694**	**6.0549**

**Table 5:** Experimental results of ablation using iterative enhancement methods.

Enhancement Method	DICM	VV	Fusion	MEF	LIME
Single parameter matrix	14.1271	16.7804	12.6768	10.8026	7.6790
Multi-parameter matrix	**5.1007**	**4.1534**	**5.4694**	**6.0549**	**5.5248**

**Table 6:** The ablation experimental results of gating mechanism.

Gating Mechanism	DICM	VV	Fusion	MEF	LIME
relu	6.8462	5.6432	6.2135	7.0024	6.1254
gelu	5.5640	5.5238	5.6898	6.2648	5.9897
hardswish	**5.1007**	**4.1534**	**5.4694**	**6.0549**	**5.5248**

## Data Availability

The data that support the findings of this study are available from the corresponding author upon reasonable request.
